# Co-circulation of two Alphaviruses in Burkina Faso: Chikungunya and O’nyong nyong viruses

**DOI:** 10.1371/journal.pntd.0011712

**Published:** 2024-06-13

**Authors:** Bachirou Tinto, Brice Bicaba, Thérèse Samdapawindé Kagoné, John Kayiwa, Ingrid Rabe, Corinne Simone Collette Merle, Alidou Zango, Ahidjo Ayouba, Sara Salinas, Dramane Kania, Yannick Simonin

**Affiliations:** 1 Pathogenesis and Control of Chronic and Emerging Infections, INSERM, University of Montpellier, Montpellier, France; 2 Centre MURAZ, Institut National de Santé Publique, Bobo-Dioulasso, Burkina Faso; 3 Centre des Opérations de Réponse aux Urgences Sanitaires, Ouagadougou, Burkina Faso; 4 Uganda Virus Research Institute, Republic of Uganda; 5 Special programme for research and training in Tropical disease (TDR), World Health Organization, Geneva, Switzerland; 6 Recherches translationnelles sur le VIH et maladies infectieuses, Université de Montpellier, IRD, Inserm, France; Faculty of Science, Ain Shams University (ASU), EGYPT

## Abstract

**Background:**

Chikungunya virus (CHIKV) and O’nyong nyong virus (ONNV) are phylogenetically related alphaviruses in the Semliki Forest Virus (SFV) antigenic complex of the *Togaviridae* family. There are limited data on the circulation of these two viruses in Burkina Faso. The aim of our study was to assess their circulation in the country by determining seroprevalence to each of the viruses in blood donor samples and by retrospective molecular and serological testing of samples collected as part of national measles and rubella surveillance.

**Methodology/Principal findings:**

All blood donor samples were analyzed on the Luminex platform using CHIKV and ONNV E2 antigens. Patient samples collected during national measles-rubella surveillance were screened by an initial ELISA for CHIKV IgM (CHIKjj Detect IgM ELISA) at the national laboratory. The positive samples were then analyzed by a second ELISA test for CHIKV IgM (CDC MAC-ELISA) at the reference laboratory. Finally, samples that had IgM positive results for both ELISA tests and had sufficient residual volume were tested by plaque reduction neutralization testing (PRNT) for CHIKV and ONNV. These same patient samples were also analyzed by rRT-PCR for CHIKV. Among the blood donor specimens, 55.49% of the samples were positive for alphaviruses including both CHIKV and ONNV positive samples. Among patient samples collected as part of national measles and rubella surveillance, 3.09% were IgM positive for CHIKV, including 2.5% confirmed by PRNT. PRNT failed to demonstrate any ONNV infections in these samples. No samples tested by RT-qPCR. had detectable CHIKV RNA.

**Conclusions/Significance:**

Our results suggest that CHIKV and ONNV have been circulating in the population of Burkina Faso and may have been confused with malaria, dengue fever or other febrile diseases such as measles or rubella. Our study underscores the necessity to enhance arbovirus surveillance systems in Burkina Faso.

## Author summary

Chikungunya virus (CHIKV) and O’nyong nyong virus (ONNV) are transmitted to humans by the bite of mosquitoes; CHIKV by genus *Aedes* genus and ONNV by *Anopheles* genus mosquitoes. Diseases caused by these two viruses can be confused with other common infectious pathologies including malaria, dengue fever, measles, and rubella, Prior to 2023, there were limited data on the circulation of CHIKV and ONNV in Burkina Faso. The aim of our study was to assess their circulation in the country by determining their seroprevalence in blood donor samples and by carrying out retrospective molecular and serological tests on samples collected as part of national measles and rubella surveillance. Among the blood donor specimens, 56.48% of samples were IgG-positive for CHIKV and 54.49% were IgG-positive for ONNV. Among patient samples collected as part of national measles and rubella surveillance, 3.09% were IgM positive for CHIKV, including 2.5% confirmed by plaque reduction neutralizing test (PRNT). Our results suggest that CHIKV and ONNV circulated in the population of Burkina Faso and could have been misdiagnosed clinically as malaria, dengue fever or other febrile diseases such as measles or rubella. These results underscore the necessity to enhance arbovirus surveillance systems in the country through appropriate laboratory testing.

## Introduction

Chikungunya virus (CHIKV) and O’nyong nyong virus (ONNV) are phylogenetically related alphaviruses of in the Semliki Forest Virus (SFV) antigenic complex of the *Togaviridae* family sharing 90% nucleotide sequence homology [1,2]. CHIKV is transmitted to humans by the bite of infected *Aedes* genus mosquitoes, with *Aedes aegypti* and *Aedes albopictus* being the main vectors that maintain the urban transmission cycle and humans are the amplifying host [3]. ONNV is transmitted by the bite of infected *Anopheles* genus mosquitoes, ONNV is mainly by *Anopheles funestus* and *Anopheles gambiae* which are also malaria vectors [4]. Contrary to *Aedes spp*, with diurnal activity, *Anopheles* have a nocturnal activity, taking their blood meals at night and thus prevention of CHIKV and ONNV infections differs with regard to vector control and mosquito bite prevention [5].

CHIKV was first isolated in 1953 in Tanganyika, which is now the United Republic of Tanzania [6]. The name chikungunya comes from the Makonde language and means "that which is bent" in reference to the posture adopted by diseased patients due to their severe joint pain [7]. About 75% of infected people develop symptoms, mainly fever, arthralgia and rash. [8]. Prolonged joint symptoms have also been reported; disabling joint pain and myalgia can persist for months or potentially extend to years [9,10]. Some patients are at higher risk of severe disease and include the elderly, neonates infected during or soon after delivery, and immunocompromised patients, who can develop meningoencephalitis, hepatitis, respiratory failure, and shock. [11]. Increased rates of Guillain-Barré syndrome have also been reported during CHIKV epidemics [12]. Since initial identification, CHIKV has caused numerous epidemics globally and autochthonous transmission has been reported on all continents except Antarctica [13]. Since the 2000s, there has been an increase in the number and scale of epidemics in Africa [14]. Serological and/or molecular evidence of CHIKV infection has been reported from 33 African countries including several West African countries such as Benin, Ivory Coast, Guinea, Nigeria, Senegal, Seychelles, Sierra Leone [14,15]. The majority of CHIKV outbreaks recorded in Africa are associated with East/Central/South African (ESCA) genotypes; however, outbreaks of West African CHIKV genotype have been documented in several West African countries [13].

ONNV was first identified in Uganda in 1959 during an epidemic that affected over 2 million people in several East African countries [16]. ONNV infection has a similar symptomatology to CHIKV. Infected individuals experience fever, joint pain (large joints), headache, rash, lymphadenitis and conjunctivitis. Hemorrhagic signs including gingivorrhagia and epistaxis have been observed on rare occasions [1,17,18]. The virus is known to be endemic in sub-Saharan Africa and has typically followed a pattern of causing large outbreaks interspersed with periods of absence when viral transmission is presumed to continue in enzootic cycles [1]. Between 1959 and 1962, countries including Uganda, Kenya, Tanzania, Malawi, and Mozambique reported outbreaks of ONNV [1]. From 1962 onwards, no ONNV epidemics were recognized until 1996, when it caused an epidemic in Uganda [18]. In West Africa, serological and molecular evidence of ONNV circulation has been reported in Nigeria, Ghana and Sierra Leone [1]. In 2013, Ivory Coast reported an outbreak among Liberian refugees [19].

In 2023, Burkina Faso reported the first recognized outbreak of chikungunya and as of December 31, 2023, a total of 311 cases have been reported (Epidemiological situation weekly, week n°6, 2024, Ministry of Health and public hygiene of Burkina Faso). Sequencing virus obtained during the outbreak investigation resulted in the identification of a new clade of the West African lineage [20]. There remains, however, a scarcity of data regarding the prior incidence and prevalence of the disease in the country that would improve understanding of potential underlying population immunity. A single study conducted on samples collected in 2015 in the capital of Burkina Faso (Ouagadougou) showed a seroprevalence of CHIKV of 29% although confirmatory cross-neutralization testing was not performed on the specimens evaluated. Furthermore, although there has been recognized regional transmission of ONNV in the past, there are no published data for ONNV in the country [21]. The objective of our study was to assess the circulation of these two viruses in Burkina Faso by determining their seroprevalence in blood donor samples and by performing retrospective molecular and serological testing of specimens collected as part of measles and rubella surveillance in Burkina Faso.

## Methods

### Ethics statement

The study was approved by the Burkina Faso Health Research Ethics Committee (2020-3-049) and was conducted in accordance with the rules described in the Declaration of Helsinki. Written and verbal consent has been obtained from patients and blood donors. The measles/rubella protocol received the approval of the Institutional Bioethics Committee of the National Center for Research and Training on Malaria under No. 2019/000006/MS/SG/CNRFP/CIB.

### Samples

#### -Blood donors

A total of 501 blood donor samples were collected from June to July 2020 in the regional blood transfusion centers of Ouagadougou and Bobo-Dioulasso, the two largest cities in Burkina Faso. The cohort included 114 (22.75%) females (median age: 28 years; interquartile range (IQR): 24–35.75 years) and 387 (77.25%) males (median age: 28 years; IQR 24–35 years). Collected samples were centrifuged and stored at -80°C at the Centre MURAZ, Institut National de Santé Publique (INSP) in Bobo-Dioulasso before laboratory analysis.

#### -Patient serum samples

Overall, 2,588 patient samples collected nationwide between 2007 and 2019 for measles-rubella testing as part of national surveillance in Burkina Faso and stored at -80°C in the biobank of the MURAZ center in Bobo-Dioulasso were used for molecular and serological testing for CHIKV, dengue and Zika viruses. This testing was conducted as part of a multi-country evaluation of the use of measles-rubella surveillance specimens for integrated *Aedes*-borne arbovirus surveillance and the findings of the full evaluation will be published separately. Samples were collected according to the definition of a suspected measles case, namely, anyone with fever, a generalized maculopapular (non-vesicular) rash and cough or/a cold or conjunctivitis (red eyes), or anyone in whom the clinician suspects measles. This sample set included sera from 1,137 (43.93%) females (median age: 7 years; interquartile range (IQR): 2–14 years) and 1,451 (56.07%) males (median age: 7 years; IQR: 2–19 years).

### Sandwich Enzyme-Linked Immunosorbent Assay

Testing at the Centre Muraz was performed using the CHIKjj Detect IgM ELISA from InBios international (Seattle, Washington, USA) for the detection of anti-CHIKV antibodies in patient specimens collected for measles-rubella surveillance. The CHIKjj Detect IgM ELISA test is an enzyme-linked sandwich immunoassay for the detection of human IgM antibodies targeting CHIKV E2/E1 envelope glycoproteins. However, this test may cross-react with other alphaviruses such as ONNV and therefore a serum neutralization test is required to confirm positive ELISA results. The test was performed according to the manufacturer’s procedure and instructions.

### CDC MAC-Enzyme-Linked Immunosorbent Assay

Samples were subsequently tested at the Uganda Virus Research Institute, serving as a reference laboratory for the evaluation, by IgM ELISA with CHIKV viral antigens as previously described [22]. Ratios of patient optical density values to negative control values (P/Ns) were calculated for IgG and IgM ELISAs. Values >3 were considered positive, and values 2–3 were considered equivocal.

To optimize the use of limited resources, IgM testing was first repeated at the reference laboratory to determine concurrence with the IgM findings at the national laboratory using the standard IgM reference test (CDC MAC-ELISA). For samples where there was concurrence, PRNT was performed. This allowed observation of agreement between the assays and also to alleviate the burden of the time and resource intensive PRNTs by restricting the test to those samples more likely to be true positives.

### Plaque reduction neutralizing reference test (PRNT)

A plaque reduction assay was performed as previously described [23] was used. Briefly, Vero-cells at a concentration of 65000cells/ml were seeded into 6-well plates (GreinerBio-One GmbH, Germany) at a volume of 3 ml/well. Cells were cultured in growth media (1X Eagle’s Minimum Essential Medium-ATCC, 8% heat inactivated fetal bovine serum (FBS), 100 units Penicillin-Streptomycin, Gentamycin 50 mg/ml and Fungizone 1 mg/ml) at 37°C with 5% CO_2_ for 3 to 4 days. After incubation, media was removed from the cell monolayer by dumping. All test sera were inactivated at 56°C, for 30 min to remove complement factor before serially diluting them two-fold ranging from 1:10 to 1:20480 for the final dilutions in BA-1 diluent (10X M199 Hanks’ Salts without L-Glutamine, 5% Bovine Serum Albumin, 1M TRIS-HCL pH 7.5, L-Glutamine, 7,5 Sodium Bicarbonate, 100X Penicillin-Streptomycin, 1000X Fungizone dissolved sterile water). The test sera were mixed with approximately 200 PFU of the reference CHIKV R92082 and ONNV UG MP303 virus and 0.1 ml of virus-serum mixture was added to confluent monolayer of cells per well and incubated at 37°C with 5% CO_2_ for 1 hour. The first overlay medium (consisting of Miller’s 2X Ye-Lah - 10X Earle’s Buffered Salts Solutions, KCL, NaCl, NaH_2_PO_4_-H_2_O, Glucose, CaCl_2_-H_2_O, MgCl_2_-6H_2_0 dissolved in distilled water), basic Ye_Lah medium (Yeast Extract-Lactalbumin hydrolysate), 2% fetal bovine serum, 1000X Fungizone, 1000X Gentamycine dissolved in sterile water) and 2% low-melting agarose (SeaKeam LE, Lonza) was added, 3 ml per well and allowed to solidify for 30 minutes at room temperature. The plates were incubated at 37°C with 5% CO_2_ for 1 day. Neutral red staining was performed by adding a neutral red (Sigma) second overlay (same as above), 2 ml per well and allowed to solidified for 30 minutes at room temperature. Plates were incubated at 37°C in 5% CO_2_ and plaques counted for 2 days after the second overlay to establish 90% neutralization titers. Back titration plates were established to ensure infectivity of cell monolayer and standardization of virus to 200 PFU/0.1 ml. NT titres < 1:10 were considered negative. Titres of 1:10 were interpreted as borderline. The PRNT antibody titres presented refer to the reciprocal of the last serum dilution that reduced by 90% (PRNT-90) the number of virus plaques cell clusters infected by 100 Plaque Forming Units/0.1 ml of the reference CHIKV R92082 and ONNV UG MP303 virus preparation.

### Luminex

All blood donor samples were analyzed using Luminex technology and CHIKV and ONNV E2 antigens following the procedures as previously described [24]. Recombinant CHIKV and ONNV E2 proteins were coupled to Luminex beads to detect Immunoglobulin G (IgG) directed against these antigens. Samples were diluted 1:200, placed in the presence of the beads and incubated overnight at 4°C. The fluorescence intensities of the antigen-antibody reactions are read with the Bioplex 200 device. The E2 antigen used for CHIKV has a sensitivity of 88.89% and a specificity of 98.48% and that for ONNV has a specificity of 96.97%. The sensitivity of the viral antigen used for ONNV could not be determined due to the absence of a positive control.

### rRT-PCR CHIKV

Patient samples collected during measles-rubella surveillance were analyzed by rRT-PCR for CHIKV. Nucleic acids were extracted from human sera using the QIAamp Viral Mini manual extraction kit (Qiagen, Hilden, Germany). Amplification was performed with the Roche LightCycler 480 using the U.S. Centers for Disease Control and Prevention (U.S. CDC) Trioplex Real-time RT-PCR Assay according to procedures described by Santiago and al [25]. This kit allows the qualitative and specific detection of viral RNA of Zika, dengue and CHIKV viruses in a single PCR reaction.

**Fig 1:**
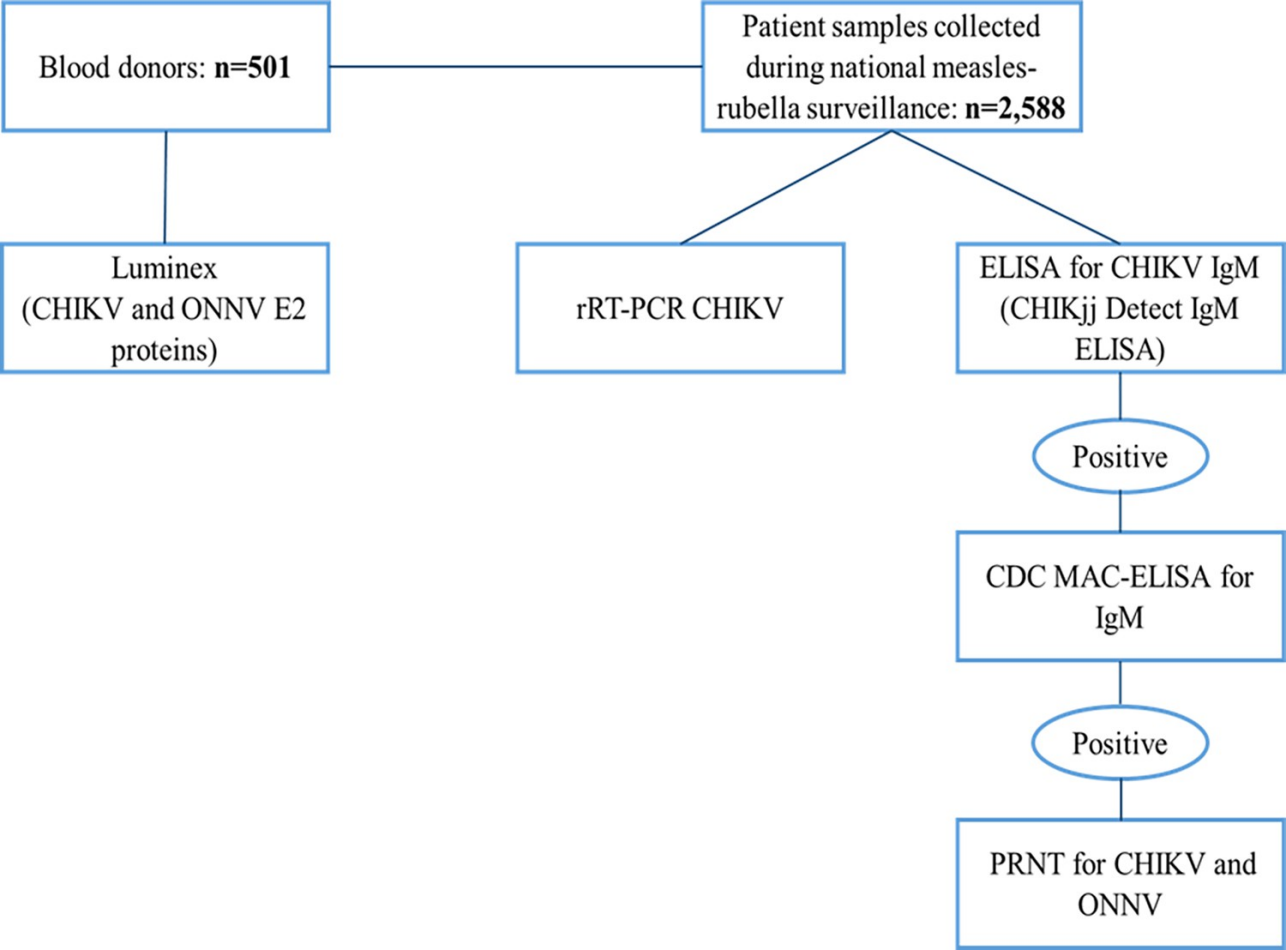
Summary of the methodological approach for samples screening.

The methodological approach is summarized in Fig 1.

### Statistical analysis

The correlation between seroprevalence and independent variables such as geographic collection site, gender, and age of blood donors was analyzed using the Pearson chi-square test or Fisher’s exact test. We also used the Odds ratio to show the association or not of independent variables with seroprevalence. Figures were made using Excel 2016, version 2302 and maps were made using Datawrapper online software.

## Results

### Serological Screening for CHIKV and ONNV in blood donors

We performed Luminex analysis on 501 blood donor samples collected in 2020 at two regional blood transfusion centers of Burkina Faso to assess the circulation of CHIKV and ONNV in the country (Table 1). We detected CHIKV-IgG in 283 samples and ONNV-IgG in 273 samples. In 223 persons we detected antibodies to both CHIKV and ONNV (44.5% CI95%: 40.21–48.88). This represents 278 samples positive for alphaviruses with a total seroprevalence of 55.49% (CI95%: 51.14–59.84).

**Table 1:** Luminex results for CHIKV and ONNV in blood donor. N = 501.

Variable	Luminex n (%)		
	CHIKV+	ONNV+	CHIKV+ONNV+
**Total**	283 (56.48)	273 (54.49)	223 (44.5)
**Origin**			
Ouagadougou	142 (55.46)	134 (52.34)	112 (43.75)
Bobo-Dioulasso	141 (57.55)	139 (56.73)	111 (45.30)
**Gender**			
Male	233 (60.20)	223 (57.62)	186 (48.06)
Female	50 (43.85)	50 (43.58)	37 (32.45)
**Age**			
18–24	79 (46.47)	82 (48.23)	61 (35.88)
25–34	115 (58.37)	113 (57.36)	92 (46.70)
35–44	62 (65.26)	55 (57.89)	47 (49.47)
45–59	27 (69.23)	23 (58.97)	23 (58.97)

Statistical analyses showed a significant difference between the sex and age of blood donors and CHIKV seroprevalence (Table 2). Males were more exposed than females and CHIKV seroprevalence increased with age; individuals aged 35 years and older had higher seroprevalences. A statistically significant difference was also observed between the sex of blood donors and the seroprevalence of ONNV as we detected a higher seroprevalence in males than in females (Table 2).

**Table 2:** CHIKV and ONNV seroprevalence according to the socio-demographic characteristics of the blood donors.

Variable	CHIKV			ONNV		
	Positive n (%)	Odds ratio IC95%	P-value	Positive n (%)	Odds ratio IC95%	P-value
**Origin**			0.6531			0.3695
Ouagadougou	142/256 (55.46)	1		134/256(52.34)	1	
Bobo-Dioulasso	141/245 (57.55)	1.09 [0.77–1.55]		139/245(56.73)	1.19[0.84‐1.69]	
**Gender**			0.0025*			0.0103*
Male	233/387 (60.20)	1		223/387 (57.62)	1	
Female	50/114 (43.85)	0.52 [0.34–0.79]		50/114 (43.58)	0.57 [0.37–0.87]	
**Age**			0.0051*			0.2512
18–24	79/170 (46.47)	1		82/170(48.23)	1	
25–34	115/197 (58.37)	1.62 [1.07–2.45]		113/197(57.36)	1.44[0.95‐2.18]	
35–44	62/95 (65.26)	2.16 [1.29–3.63]		55/95(57.89)	1.28[0.89‐2.46]	
45–59	27/39 (69.23)	2.59 [1.23–5.45]		23/39(58.97)	1.54[0.76‐3.12]	

* p < 0.05

### Serological and Molecular Screening for CHIKV in residual measles/rubella surveillance samples

Measles and rubella are viral infections that can have a similar symptomatology to CHIKV including fever, joint pain, and rash. Thus, these diseases can be confused clinically with CHIKV or other arboviral diseases.

**Fig 2:**
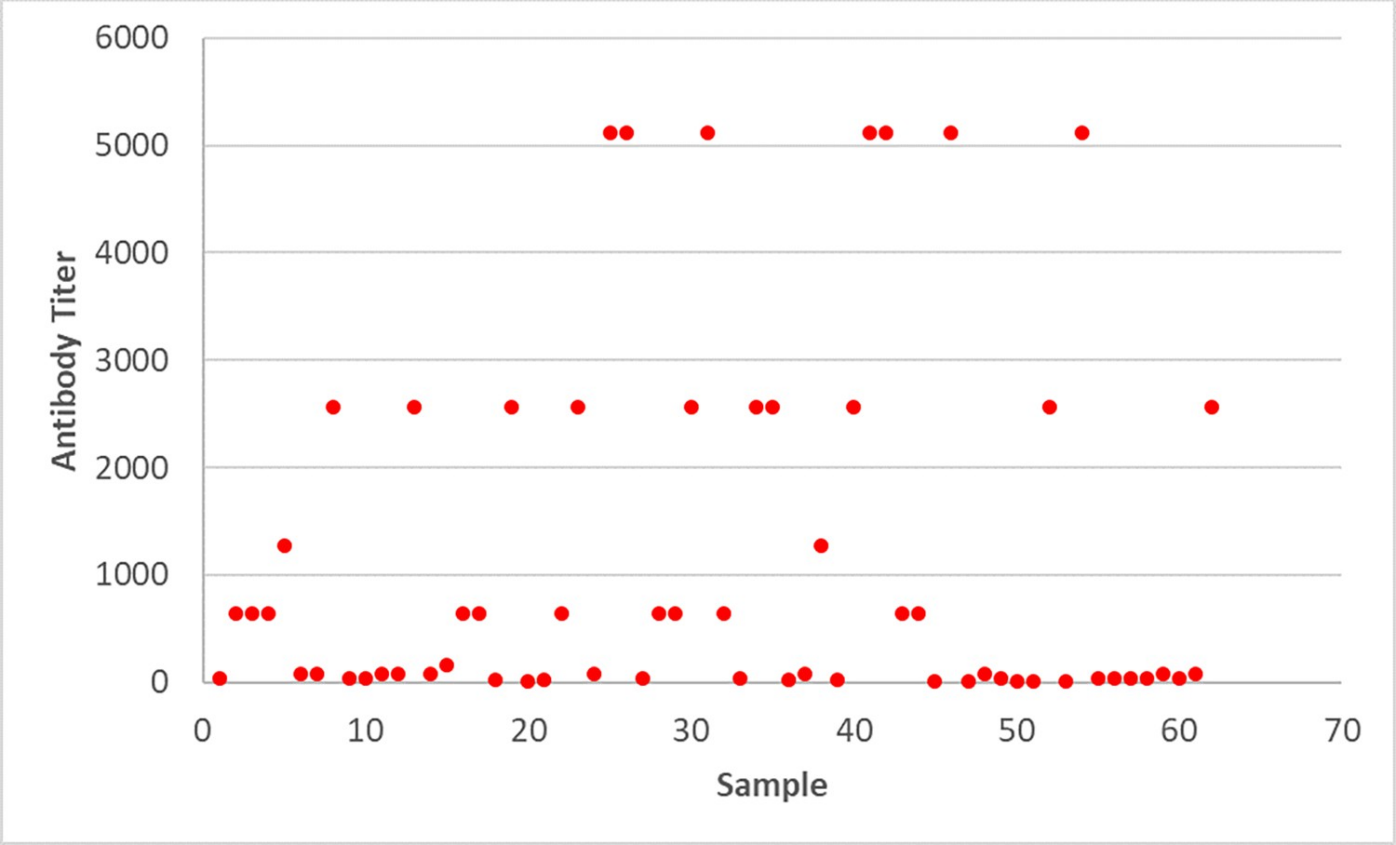
Anti-CHIKV antibody titer in patient samples after PRNT tests.

Patient samples collected during national measles-rubella surveillance were screened by an initial ELISA for CHIKV IgM (CHIKjj Detect IgM ELISA) at the national laboratory (Table 3). We detected CHIKV-IgM in 126 specimens (4.86% CI95%: 4.0–5.7). Eighty-five of these positive specimens were then analyzed by the CDC MAC-ELISA and 75 had detectable CHIKV-IgM (2.90% CI95%: 2.3–3.5). Among those, 62 samples had sufficient residual volume for testing by PRNT for CHIKV and ONNV and in all of those we confirmed CHIKV as the infecting virus (2.39% of all specimens CI95%: 1.8–3.0) with neutralizing antibody titers up to 1:5120 (Fig 2). PRNT failed to detect any ONNV neutralizing antibodies in these samples.

**Table 3:** ELISA and PRNT results in patient samples from measles/rubella surveillance. N = 2588.

Variable	CHIKjj Detect IgM ELISA n%	CHIKV IgM CDC MAC-ELISA n (%)	PRNT Positive CHIKV n (%)
**Total**	126 (4.86)	75 (2.90)	62 (2.39)
**Gender**			
Male	78 (5.37)	48 (3.31)	43 (2.96)
Female	48 (4.22)	27 (2.37)	19 (1.67)
**Age**			
0–5	18 (1.61)	7 (0.62)	4 (0.36)
6–20	55 (5.67)	33 (3.40)	28 (2.88)
21–30	42 (12.80)	28 (8.54)	23 (7.01)
31–40	8 (6.25)	5 (3.90)	5 (3.91)
41–60	3 (6.67)	2 (4.44)	2 (4.44)

**Fig 3:**
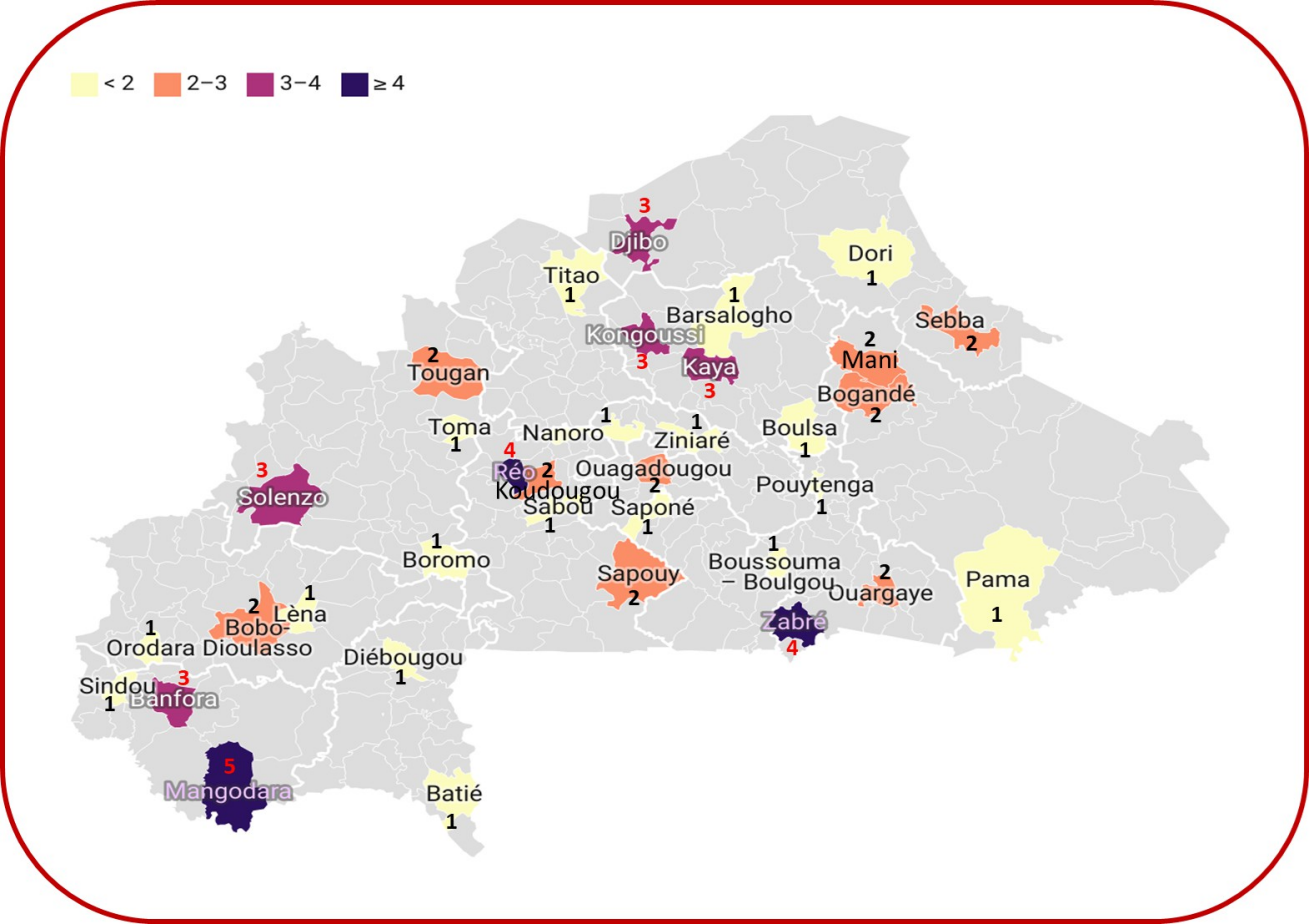
Geographical distribution of positive patient for antibodies against CHIKV: The numbers represent the number of PRNT-confirmed CHIKV infections. (Figure was made with BioRender.com).

The samples originated from different areas within Burkina Faso, and the quantity of samples testing positive for anti-CHIKV antibodies differed across these regions. Particularly, a significant proportion of positive samples was observed in the southwestern region (Fig 3).

No CHIKV RNA was detected in any specimens tested by rRT-PCR.

## Discussion

The evaluations reported describe CHIKV and ONNV testing in two different sample populations in Burkina Faso namely patients with febrile rash illness, detected through national measles rubella surveillance, and blood donors who were asymptomatic at the time of donation. In addition, the methods differed with the testing for the former, indicative of recent infection by IgM whereas the testing among blood donors was for prior infection by IgG. Among the measles-rubella specimens, we identified 75 (%) with evidence of CHIKV infection by IgM, including 62 (%) confirmed by cross-neutralizing PRNT with detectable titers to CHIKV and not to ONNV among the samples tested probably because the samples were previously selected with an ELISA test considered specific for CHIKV.

Among blood donors we detected antibodies to CHIKV and ONNV in over 50% of specimens tested. The Luminex test conducted on these samples detects all categories of IgG antibodies, irrespective of whether they possess neutralizing properties or not which allows us to have a broader view of the presence of antibodies in these samples. Furthermore, IgG would suggest infection at any time prior to specimen collection and thus would be expected to be detectable in a higher percentage of patients than those tested for acute infection. It should also be noted that it is possible to have cross-reactions between CHIKV and ONNV, which can only be excluded by serum neutralization.

There have been very few studies on the seroepidemiology of CHIKV in Africa. A study published in 2022 reported a seroprevalence of CHIKV of 29% in samples collected in 2015 from the general population in Ouagadougou (the capital of Burkina Faso); however, no gold standard neutralizing antibody confirmatory testing was performed [21]. Serological and/or molecular evidence of CHIKV circulation has been reported in more than 30 African countries, including Ivory Coast and Benin, two countries bordering Burkina Faso with which there is extensive trade in goods and people [14,15]. Two CHIKV seroprevalence studies conducted in Cameroon between 2000–2003 and in 2007 reported results similar to our own among blood donors: 46.5% and 49.5% respectively [26,27]. Another study conducted in 2007 in Benin among pregnant women reported a CHIKV seroprevalence of 36.2% [28]. Our results suggest that CHIKV has been circulating in the population and may have been misdiagnosed as malaria, dengue or other febrile illnesses such as measles or rubella, which circulate in Burkina Faso and share similar clinical features to disease caused by both CHIKV and ONNV. For IgM CHIKV detected in patient samples, the Mangodara area recorded the most positive cases. This could be explained by the fact that it is a forest area with high rainfall compared to other regions of the country and therefore probably with a high density of vectors. This area is located on the border with Ivory Coast, where there is evidence of CHIKV circulation [14,15]. The lack of detection of CHIKV RNA by rRT-PCR in the measles-rubella surveillance specimens may be attributable to the short period of detectable viremia or to the prolonged storage of the specimens tested that might have resulted in degradation of viral genetic material [29]. In Burkina Faso, patients consult hospitals late and therefore probably outside the viremic window; this makes the detection of viruses with a short viremia such as CHIKV difficult by RT-PCR. However, the presence of CHIKV IgM observed in fever samples shows that these are recent infections that occurred less than 3 months ago. Indeed, during CHIKV infection, IgM antibodies appear around the fifth day of infection and can persist for up to 3 months. IgG antibodies appear later around the seventh and tenth day of the disease and persist for several years [11].

The ONNV has been detected in two countries bordering Burkina Faso: Ghana and Ivory Coast with an epidemic in 2013 [1,16], but the presence of ONNV in Burkina Faso had not been established prior to our study. ONNV probably circulates less in Africa than CHIKV. However, we believe that this circulation is largely underestimated due to the lack of virus surveillance systems in the different countries as highlighted in our study. Moreover, ONNV infection has a similar symptomatology to CHIKV and other diseases and may go unrecognized. Furthermore, there is a lack of commercially available kits for both molecular and serological tests, and our understanding of the kinetics of biological markers during an ONNV infection remains limited making it difficult to monitor this virus [30]. In our study we identified 33.6% co-carriage of anti-CHIKV and anti-ONNV antibodies in blood donors. A previous study carried out in Kenya had also reported a co-circulation of CHIKV and ONNV with a co-carriage of anti-CHIKV and anti-ONNV antibodies of 38% [31]. Seroneutralization tests may help in confirming antibody specificity and differentiating prior infection between the two viruses. In our study, we were unable to carry out seroneutralization tests on specimens that were either IgG-positive to ONNV alone or with detectable anti-CHIKV and anti-ONNV IgG antibodies. We found a statistically significant difference between the gender of blood donors and CHIKV and ONNV seroprevalences with a higher seroprevalences in males. The lifestyle of the Burkinabe population could explain this difference for CHIKV. Indeed, in Burkina Faso, men have the culture of meeting for discussions in the evening after work on outdoor terraces, thus exposing themselves to a greater risk of mosquito bites, unlike women who return home after work to perform household chores. A study conducted during the 2019 CHIKV outbreak in the Democratic Republic of Congo also showed that CHIKV prevalence increased with age [32]. Similar results have been observed with CHIKV in Bangladesh [33]. This explanation is not applicable to ONNV since the vectors of the two viruses differ. CHIKV is transmitted by the bite of *Aedes* mosquitoes, which are active during the day and bite mostly in the morning and late afternoon, while ONNV is transmitted by the bite of the *Anopheles* mosquito, primarily active during the nighttime hours [1,5]. A socio-anthropological study would be interesting to better understand this disparity in risk of infection by gender. However, it should be noted that our results differ from those of Kyungah Lim et al who found that in Ouagadougou (capital of Burkina Faso), women were about 1.4 times more likely to be infected with CHIKV than men [21]. A statistically significant difference was also observed between blood donor age and CHIKV seroprevalence. People aged 35 years and older had higher seroprevalences. This age group represents the most active people in society and are most often at risk. A similar discovery was observed in Burkina Faso with a different arbovirus, the Zika virus (ZIKV), where the seroprevalence among blood donors escalated with advancing age [34].

In conclusion, our study revealed the active circulation of alphaviruses in Burkina Faso. Further investigations are required to distinguish the strains of these circulating viruses and better understand their impact on human health. Our study underscores the necessity to enhance arbovirus surveillance systems in Burkina Faso.

### Disclaimer

I. Rabe and CSC Merle are currently staff members of the World Health Organization; the authors alone are responsible for the views expressed in this publication and they do not necessarily represent the decisions, policy or views of the WHO.
